# Discrepancy in the perception of symptoms of cognitive decline between older adults and their family members: results of the Toyama dementia survey

**DOI:** 10.1186/s12883-019-1581-2

**Published:** 2019-12-26

**Authors:** Nobue Nakahori, Michikazu Sekine, Masaaki Yamada, Takashi Tatsuse, Hideki Kido, Michio Suzuki

**Affiliations:** 1 Faculty of Nursing Science, Tsuruga Nursing University, 78-2-1 Kizaki, Tsuruga, Fukui, 914-0814 Japan; 2 Department of Epidemiology and Health Policy, Graduate School of Medicine and Pharmaceutical Sciences, University of Toyama, 2630 Sugitani, Toyama, Toyama 930-0194 Japan; 3 Kiseikai, Kido Clinic, 244 Honoki, Imizu, Toyama, 934-0053 Japan; 4 Department of Neuropsychiatry, Graduate School of Medicine and Pharmaceutical Sciences, University of Toyama, 2630 Sugitani, Toyama, Toyama 930-0194 Japan

**Keywords:** Cognitive decline, Family, Older adults, Perception, Subjective memory complaints

## Abstract

**Background:**

Early consultation is important to delay the onset of dementia. The present study aimed to explore the reasons for delaying a consultation of dementia while focusing on the differences in the perception of cognitive decline between older adults and their family members.

**Methods:**

A group of 663 older adults aged ≥65 years and living with family members in Toyama Prefecture was surveyed. The questionnaires included items that measured changes in cognitive function noticed by older adults and their family members, and the Revised Hasegawa Dementia Scale (HDS-R). The degrees of consistency on the perception of mental changes that accompanied cognitive decline were measured using the Kappa statistic.

**Results:**

Both older adults and their family members were well aware of “forgetfulness” as a symptom of cognitive decline. Only the perception of “loss of appetite” at the late stage of cognitive decline was consistent between older adults and their family (κ = 0.707). When older adults often noticed their own forgetfulness, their mean HDS-R score was 22.7, whereas that of the family members was 14.7. The combinations of perception of forgetfulness by older adults and their family members, and the mean HDS-R scores were unaware/unaware (mean HDS-R score = 27.0), aware/unaware (mean HDS-R score = 24.9), aware/aware (mean HDS-R score = 15.5), and unaware/aware (mean HDS-R score = 13.0).

**Conclusions:**

There were discrepancies in the perception of cognitive decline between older adults and their family members. Cognitive decline had progressed by the time that family members had noticed the symptom of forgetfulness in their older adult relatives. The perception gap regarding cognitive decline deters consultation of dementia.

## Background

Japan’s population is aging with an unprecedented speed compared with other world regions. The incidence of dementia is estimated at approximately one in seven Japanese ≥65 years of age, and the prevalence is projected to increase to approximately one in five by the year 2025 [1]. As there is no effective treatment for dementia at present, delaying the onset of dementia is imperative. The Lancet Committee reported that interventions to delay the onset of dementia by one year may reduce the diagnosis of dementia worldwide by nine million by the year 2050 [2]. People with mild cognitive impairment have more possibilities to revert in comparison to those with advanced cognitive impairment [3]. This encourages both the family and older adults to receive appropriate treatment at an early stage and taking appropriate measures to prepare for symptom progression.

Early consultation is necessary to prevent and delay the onset of dementia. However, cognitive decline often progresses in older adults after consultation with a doctor about dementia [4]. Self-awareness decreases with the progression of cognitive decline, which is accompanied with a lower likelihood that older adults will seek medical consultation of dementia and long-term care services, even when encouraged [5]. The effect of treatment at the late stages of cognitive decline is limited [6, 7]. It is difficult for many families to accompany their elderly relatives with suspected dementia to consultation [8].

We examined the perception of cognitive decline by older adults themselves and their cohabitating family members to explore the reasons for the delay in consultation of dementia. Some studies have analyzed the symptoms of cognitive decline in older adults [9, 10], whereas few have investigated differences in the perception of cognitive decline between older adults and their family members who live together.

The present study aimed to explore the reasons for the delay in consultation of dementia, especially differences in perceptions at each stage of cognitive decline between older adults and their family members, as clarifying the reasons for the delay in consultation of dementia may contribute to delaying the onset of dementia.

## Methods

### Participants

A 0.5% random sample of the 307,582 residents of Toyama Prefecture who were ≥ 65 years of age on October 1, 2013, was identified using computers to search a basic resident register. Of 1537 elderly individuals living at home or in elderly care facilities, 1303 (84.8%) agreed to participate in this study. Public health nurses interviewed older adults and their family members, including husbands, wives, sons, daughters, children’s spouses, and grandchildren. The person who was most familiar with the older adult among the family members responded to the questionnaire. The staff of the institution housing the older adults was also interviewed. The interviews were completed between June and August 2014 at the place of residence. Of the 1153 people who responded to all of the questions, 415 who were living unaccompanied, 30 who were institutionalized, and 45 who were separated from their family members were excluded from analysis. The remaining 663 older adults and their family members were included in the study. The characteristics of the participants are shown in Table 1.

**Table 1:** Characteristics of the 663 older adults

	*n*	%
Sex		
Men	329	49.6
Women	334	50.4
Age (years)		
Mean age	76.0 ± 7.59	
65–74	317	47.8
75–84	247	37.3
≥ 85	99	14.9
Family		
Husband	177	26.7
Wife	294	44.3
Son	66	10.0
Daughter	56	8.4
Child’s spouse	62	9.4
Grandchild	8	1.2
Living partner		
Spouse	215	32.4
Parents	19	2.9
Children couple	63	9.5
Children couple and grandchildren	174	26.2
Children couple and grandchildren couple	11	1.7
Children (unmarried)	125	18.9
Grandchildren couple	1	0.2
Others	55	8.3
Hasegawa Dementia Scale Score (points)		
Mean score	25.8 ± 5.80	
≤ 20	80	12.1
21–24	78	11.8
≥ 25	505	76.2

Older adults and their family members received written explanations of the study aims and gave written informed consent to participate. The study protocol was approved by the Ethics Committee of the University of Toyama (Toyama, Japan) and was performed in accordance with the tenets of the Declaration of Helsinki.

### Revised Hasegawa dementia scale (HDS-R)

The HDS-R is a nine-item questionnaire with a maximum score of 30 points [11] and is widely utilized for the screening of dementia in Japan. The HDS-R is strongly correlated with the Mini-Mental State Examination [12]. Its validity has been confirmed with a sensitivity and specificity of 0.90 and 0.82, respectively, when the cut-off point is set to 20/21 [11]. Dementia is suspected when the HDS-R score is ≤20. The average HDS-R score for healthy subjects diagnosed as not having dementia is 24.27 [11]. According to the cut-off and mean scores of the HDS-R, the subjects were assigned to one of three groups: greater than the mean (≥25 points), less than the mean (21–24 points), and suspected dementia (≤20 points).

### Mental changes noticed by older adults

Older adults were questioned about changes in 14 mental state symptoms in the previous 3 months. Items of each mental change were answered with either “YES” or “NO.” The symptoms are listed in Table 2 [13–16]. The questionnaires were completed by the family members by asking older adults about their awareness of changes.

**Table 2:** Mental changes noticed by the 663 older adults

Hasegawa Dementia Scale Score	≤20 (a)	21–24 (b)	≥25 (c)	Chi-square Test	Adjusted Residual	
	(*n* = 80)	(*n* = 78)	(*n* = 505)			
	%	%	%	*P* value	O < E	E < O
Loss of appetite	6.3	5.1	4.2	0.679		
Being unable to sleep at night	11.3	9.0	11.3	0.829		
Feeling more moody in the mornings and less so in the evenings	2.5	1.3	3.2	0.635		
Losing motivation and interest in things	17.5	10.3	6.9	0.006	c	a
Worrying about their health	16.3	9.0	6.3	0.008	c	a
Frequently feeling anxious	8.8	9.0	5.0	0.189		
Feeling restless	2.5	1.3	1.8	0.844		
Feeling compelled to do things despite not believing there is a need for them (washing hands, closing doors, checking the state of open flames, among others)	5.0	3.8	3.2	0.694		
Fluctuating mood	8.8	5.1	2.4	0.011	c	a
Sometimes wanting to die	5.0	2.6	2.6	0.475		
Becoming more forgetful	46.3	33.3	14.9	< 0.001	c	a,b

O < E, the rate of the measured value is significantly lower than the rate of the expected value; E < O, the rate of the expected value is significantly higher than the rate of the measured value

### Mental changes in older adults noticed by family

Family members answered questions about changes in 19 symptoms of the mental state of their older adult relatives during the previous 3 months. Items of each mental change were answered with either “YES” or “NO.” The symptoms have been previously described and are shown in Table 3 [13–16].

**Table 3:** Mental changes in the 663 older adults that were noticed by their family members

Hasegawa Dementia Scale score	≤20 (a)	21–24 (b)	≥25 (c)	Chi-square	Adjusted	
	(*n* = 80)	(*n* = 78)	(*n* = 505)	test	residual	
	%	%	%	*P* value	O < E	E < O
Lacking vitality and feeling depressed	10.0	5.1	2.0	< 0.001	c	a
Loss of appetite	7.5	6.4	3.0	0.074	c	
Constantly experiencing negative thoughts	11.3	9.0	4.6	0.028	c	a
Feeling restless	11.3	3.8	1.6	< 0.001	c	a
Feeling more moody in the mornings and less so in the evenings	3.8	0.0	3.2	0.260		
Worrying about their health	5.0	1.3	3.1	0.411		
Frequently consulting physicians and successively changing medical institutions	0.0	0.0	1.2	0.388		
Being unable to sleep at night	8.8	7.7	5.3	0.398		
Assuming the intentions of strangers to be bad and being convinced that things that are not fact are true	6.3	5.1	1.0	0.001	c	a
Seeing things that are not there and hearing voices	10.0	2.6	0.4	< 0.001	c	a
Becoming more forgetful	38.8	14.1	1.0	< 0.001	c	a,b
Being half awake at night making noise and moving about	5.0	1.3	0.2	0.001	c	a
Sometimes confusing night and day	17.5	5.1	0.8	< 0.001	c	a
Occasionally getting lost when going out	2.5	0.0	0.0	0.001	c	a
Becoming very stubborn and obstinate	21.3	10.3	5.0	< 0.001	c	a
Diminished consideration for others (selfishness)	25.0	7.7	2.6	< 0.001	c	a
Emotional instability	17.5	5.1	2.2	< 0.001	c	a
Preferring to stay home and being unwilling to meet other people	12.5	7.7	1.8	< 0.001	c	a
Losing motivation and interest in things	25.0	5.1	2.2	< 0.001	c	a

O < E, the rate of the measured value is significantly lower than the rate of the expected value; E < O, the rate of the expected value is significantly higher than the rate of the measured value

### Statistical analysis

All statistical analyses were performed using IBM SPSS Statistics for Windows, version 23 (IBM Corp., Armonk, NY, USA). The characteristics of older adults, such as sex, age, cohabitating family members, household composition, and HDS-R scores were reported using descriptive statistics by calculating the number and percentage of each variable. Mental changes recognized at each stage of cognitive decline were reported by calculating the percentage of positive changes. HDS-R scores of ≤20, 21–24, and ≥ 25 points were compared using the chi-square test to investigate the transition of symptoms at each stage of cognitive decline. In addition, residual analysis was performed to differentiate the stages that are significantly frequent from those that are significantly less frequent. Both family members and older adults were questioned regarding six common symptoms of cognitive decline in older adults. For these symptoms, the degree of agreement of recognition was evaluated using the Kappa statistic. The mean HDS-R scores of older adults who noticed their forgetfulness and the mean values of their family members who noticed forgetfulness of their older adult relatives were compared. The level of statistical significance was set at 5%.

## Results

The characteristics of the 663 older adults [329 (49.6%) men and 334 (50.4%) women] are presented in Table 1. The mean age of the older adults was 76.0 ± 7.59 years. The family members included 117 husbands (26.7%), 294 wives (44.3%), 66 sons (10.0%), 56 daughters (8.4%), 62 children’s spouses (9.4%), and eight grandchildren (1.2%). Living partners included 215 spouses (32.4%), 19 parents (2.9%), 63 children couple (9.5%), 174 children couple and grandchildren (26.2%), 11 children couple and grandchildren couple (1.7%), and 125 children (unmarried) (18.9%), one grandchildren couple (0.2%), and 55 others (8.3%). The HDS-R score was ≤20 points in 80 (12.1%) participants, 21–24 points in 78 (11.8%), and ≥ 25 points in 505 (76.2%).

Table 2 shows the percentages of older adults who noticed mental changes ordered by the level of cognitive decline. The mental changes accompanying each stage of cognitive decline that were noticed by the older adults themselves are presented in decreasing order. The most common changes in cognitive decline were “becoming more forgetful (14.9%)” and “being unable to sleep at night (11.3%)” in the HDS-R ≥ 25 group, “becoming more forgetful (33.3%)” and “losing motivation and interest in things (10.9%)” in the HDS-R = 21–24 group, and “becoming more forgetful (46.3%)” and “losing motivation and interest in things (17.5%)” in the HDS-R ≤ 20 group. Four of the 11 symptoms were significantly associated with the HDS-R score. Subgroup analysis revealed that these items were significantly more common in participants with HDS-R scores ≤20 points and significantly less common in those with scores ≥25 points. “Forgetfulness” was also significantly more common in participants with HDS-R scores of 21–24 points.

Table 3 shows the percentage of family members who noticed mental changes in their older adult relatives, as ordered by the level of cognitive decline. The mental changes accompanying cognitive decline that family members noticed at each stage in decreasing order were “being unable to sleep at night (5.3%)” and “becoming very stubborn and obstinate (5.0%)” in the HDS-R ≥ 25 group, “becoming more forgetful (14.1%)” and “becoming very stubborn and obstinate (10.3%)” in the HDS-R = 21–24 group, and “becoming more forgetful (38.8%),” “losing motivation and interest in things (25.0%),” and “diminished consideration for others (selfishness) (25.0%)” in the HDS-R ≤ 20 group. Of the 19 symptoms, 14 were significantly associated with the HDS-R score. Subgroup analysis revealed that these items were significantly more common in participants with HDS-R scores of ≤20 points and significantly less common in those with scores of ≥25 points. “Forgetfulness” was also significantly more common in participants with HDS-R scores of 21–24 points.

Table 4 shows the percentage of each of the four combinations of mental changes with or without older adults’ awareness and with or without their family’s awareness in each group divided into three subgroups based on the HDS-R score. Table 4 also shows the coincidence in perception of cognitive decline-related mental changes noticed by family members and those noticed by the older adults themselves. The perception of loss of appetite in older adults with HDS-R scores ≤20 points (κ = 0.707) was in substantial agreement with the perception of the cohabitating family members. There was poor agreement in the perception of symptoms, except for loss of appetite, in all three HDS-R groups between older adults and their family members.

**Table 4:** Degree of coincidence of mental changes associated with cognitive decline noticed by the 663 older adults and their family members

Hasegawa Dementia Scale Score			≤20 points (*n* = 80)			21–24 points (*n* = 78)			≥25 points (*n* = 505)		
			Family			Family			Family		
			Yes %	No %	κ statistic	Yes %	No %	κ statistic	Yes %	No %	κ statistic
Loss of appetite	Participants	Yes	5.0	1.3	0.707	3.8	1.3	0.647	2.0	2.2	0.540
		No	2.5	91.3		2.6	92.3		1.0	94.9	
Being unable to sleep at night	Participants	Yes	3.8	7.5	0.307	3.8	5.1	0.413	3.8	7.5	0.410
		No	5.0	83.8		3.8	87.2		1.6	87.1	
Losing motivation and interest in things	Participants	Yes	7.5	10.0	0.185	2.6	7.7	0.284	0.8	6.1	0.146
		No	17.5	65.0		2.6	87.2		1.4	91.7	
Worrying about their health	Participants	Yes	3.8	12.5	0.299	0.0	9.0	−0.023	1.6	4.8	0.304
		No	1.3	82.5		1.3	89.7		1.6	92.1	
Feeling restless	Participants	Yes	1.3	1.3	0.147	1.3	0.0	0.306	0.4	1.4	0.222
		No	10.0	87.5		2.6	96.2		1.2	97.0	
Becoming more forgetful	Participants	Yes	26.3	20.0	0.339	11.5	21.8	0.360	0.6	14.3	0.058
		No	12.5	41.3		2.6	64.1		0.4	84.8	

Table 5 shows the average HDS-R scores of older adults who noticed forgetfulness and those of family members who noticed forgetfulness in their older adult relatives. Table 5 also shows the average HDS-R scores for four combinations of older adults who do and do not notice their forgetfulness and their family members who do and do not notice the forgetfulness of older adults. The mean HDS-R scores of older adults and family members who noticed “becoming more forgetful” were 22.7 ± 8.00 and 14.7 ± 8.30, respectively. The combinations of perception of forgetfulness by older adults and their family members, and the mean HDS-R scores (±standard deviation, SD) were unaware/unaware (27.0 ± 3.98), aware/unaware (24.9 ± 6.53), aware/aware (15.5 ± 8.05), and unaware/aware (13.0 ± 8.94).

**Table 5:** Mean HDS-R scores of older adults and their family members who notice forgetfulness.

					*n* = 663
			Family		
			HDS-R scores, mean ± SD		
		Perception of forgetfulness	Yes	No	Older adults notice their forgetfulness
Older adults	HDS-R scores, mean ± SD	Yes	15.5 ± 8.05	24.9 ± 6.53	22.7 ± 8.00
		No	13.0 ± 8.94	27.0 ± 3.98	
		Family members notice the forgetfulness of older adults	14.7 ± 8.30		

## Discussion

In Japan, families find it difficult getting older adults suspected of having dementia to a medical consultation. Early intervention for dementia is an effective means of delaying its onset. In reality, there are few older adults who get early consultations or interventions. The present study assessed the reasons for delaying the consultation for dementia focusing on the differences in the perception of cognitive decline between older adults and their family members. The result showed that family members do not know when older adults begin to notice forgetfulness and it is unlikely that older adults see a doctor at this time. Moreover, by the time the family notices the older adult’ forgetfulness, the older adult’s cognitive decline is significant. The results of this study suggest that the ideal time for early intervention is when older adults become aware of their forgetfulness.

Older adults report that they notice mental changes at the early stage of cognitive decline [17–21]. As in previous studies, our results showed that older adults were more aware of forgetfulness than their family members at the early stage of cognitive decline. When the stage of cognitive decline progresses, older adults are likely to experience low motivation, be concerned about health, and have fluctuating mood. The family is aware of the low motivation but unaware of the concern about health. These changes are considered to be an indicator that should be noted as mental changes noticed by the older adults with cognitive decline. The symptom most recognized by older adults in any stage of cognitive decline was forgetfulness. The results of the present study indicated that self-awareness of forgetfulness is a sensitive indicator of cognitive decline, as subjective memory complaints were strongly correlated with the HDS-R score. Previous studies have reported that older people with subjective memory complaints are at a greater risk of future cognitive impairment [22–25].

As in previous studies [26, 27], our results showed that the family members of older adults in this study were aware of mental changes that occurred with each stage of cognitive decline. With the progression of cognitive decline, family members were likely to notice lower motivation, selfishness, and stubbornness of their older adult relatives. The symptom that the family members most perceived in any stage of cognitive decline was forgetfulness. However, the mean HDS-R scores of older adults who notice their own forgetfulness were greater by about eight points than that of the family members. In other words, by the time family members noticed the symptoms of forgetfulness in their older adult relatives, the stage of dementia had considerably progressed. In many cases, children of older adults may notice something unusual about their parents, but they have difficulty in recognizing such changes as cognitive decline [28]. The present study showed that it is difficult for family members to notice mental changes in older adults even if they are cohabitating.

Among older adults and their family members, the perceptions of most of symptoms of cognitive decline were discrepant. Only the perception of loss of appetite was consistent between older adults and their family members at a HDS-R score of ≤20 points. This is probably due to the family members being able to directly observe changes in daily dietary habits. However, the perceptions of symptoms were not consistent between older adults and their family members at a HDS-R score of ≥21 points. The degree of coincidence of perception of forgetfulness between older adults and family members was low. We found that family members tended not to notice forgetfulness even when older adults with normal cognitive function sensed forgetfulness, unless older adults said it. Conversely, we speculate that older adults with low cognitive function gradually lose their ability to perceive their own forgetfulness; thus, their perception begins to diverge from that of their family members, which suggests that it would be too late once their family members had noticed cognitive decline of their older adult relatives.

**Fig. 1:**
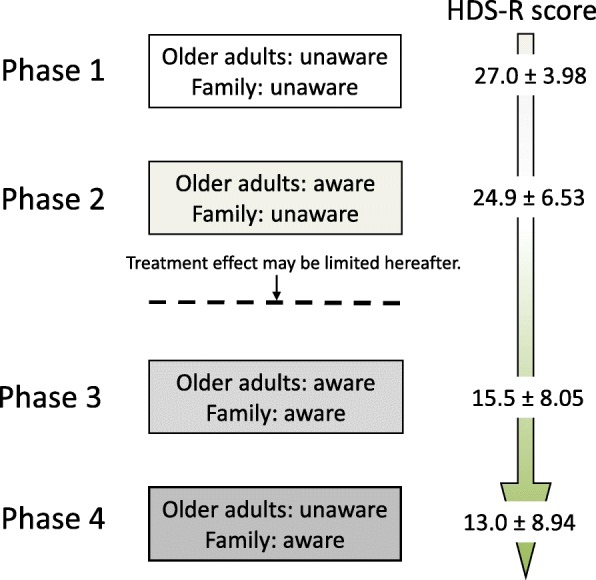
Transition of perception of forgetfulness

We depicted the four combinations of perception of forgetfulness (Fig. 1) between older adults and family members as unaware/unaware (phase 1), aware/unaware (phase 2), aware/aware (phase 3), and unaware/aware (phase 4). The average HDS-R scores for the four combinations of perception of forgetfulness of older adults/family members were in descending order. In particular, in the first phase (mean HDS-R score = 27.0), both older adults and family members are unaware of the symptom of forgetfulness, as cognitive function seems to be normal. In the second phase (mean HDS-R score = 24.9), older adults are aware of forgetfulness, whereas their family members are not. Treatment should be effective in this phase. In the third phase (mean HDS-R score = 15.5), both older adults and their family members are aware of the symptom of forgetfulness and many start to consider a consultation of dementia, but the effect of treatment may be limited. In the fourth phase (mean HDS-R score = 13.0), older adults are unaware of forgetfulness, but family are aware. As cognitive decline of older adults is progressed in this phase, and the family members may have difficulty in taking their older adult relatives to consultation of dementia. Although not everyone goes through these phases in the order shown, any preventive measures should be important, especially in the second phase.

The results of the present study indicate the importance of subjective memory complaints of older adults. Reportedly, the family members living with older adults tend to disrupt the consultation [29]. Older adults who notice their forgetfulness are less likely to consult a doctor if some of their family members regard cognitive decline as mild loss of the ability to remember facts as observed in a normal part of the aging process. Children tend to deny that their parents have dementia [28, 30]. Normalcy bias may effectively lead to the dismissal of dementia by both older adults and their family members. The present study suggested that early measures for delaying the onset of dementia would be required when older adults themselves notice their forgetfulness.

There were some limitations to this study that should be addressed. First, there are limitations to the survey conducted by families using the questionnaire. We did not closely evaluate the participation of older adults and their family members in shared daily activities. In addition, this questionnaire was not validated for reliability. This questionnaire does not cover all the mental changes associated with cognitive decline; therefore, the results need to be interpreted based on this limitation. However, it includes symptoms that are considered to be clinically important [13–16]. As the family questioned older adults about their awareness of changes, they may have influenced the perception results of the older adults. However, in this survey, the family members were instructed to read out each question to the elderly and fill in the responses exactly as they were answered; therefore, it is unlikely that the family has changed their answers. A study reported that the proxy quality of life assessment of patients with dementia tends to be lower than self-assessment [31]. This must be considered because the self-assessment in the questionnaire was completed by family members in the present study. Second, this is a cross-sectional study and does not longitudinally investigate the change in the perception of cognitive decline among older adults and their family members. However, this study showed a possibility that there are stages in the combinations of the perception of forgetfulness, which may help physicians to diagnose an early stage of dementia; thus, the results of this study may be useful in primary care settings.

## Conclusions

The results of this study showed discrepancies in the perception of most symptoms of cognitive decline between older adults and their family members. Both older adults and their family members significantly noticed forgetfulness. However, the mean HDS-R scores of older adults who noticed their own forgetfulness were greater by about eight points than those of family members. Cognitive decline progresses by the time that family members take notice of the symptom of forgetfulness of their older adult relatives. It is suggested that the perception gap shown above between older adults and their family members regarding cognitive decline works to deter consultation of dementia. Subjective memory complaints are sensitive indicators of cognitive decline. These results suggest that early measures to delay the onset of dementia are required once older adults start having concerns about forgetfulness.

## Data Availability

The datasets analyzed during the current study are available from the corresponding author upon reasonable request.

## Abbreviations

CI: Confidence interval; OR: Odds ratio
HDS-R: Revised Hasegawa Dementia Scale
